# Botox: Current and Emerging Trends for Dental Practitioners in Esthetic Dentistry

**DOI:** 10.7759/cureus.64052

**Published:** 2024-07-08

**Authors:** Ojasvi Rambole, Amit Reche, Priyanka Paul

**Affiliations:** 1 Department of Public Health Dentistry, Sharad Pawar Dental College and Hospital, Datta Meghe Institute of Higher Education and Research, Wardha, IND

**Keywords:** salivary gland disorders, gummy smile, tmj, bruxism, botulinum toxin, botox

## Abstract

There are numerous medical and dental disorders for which there are no effective traditional therapy options. For various medical and dental disorders, botulinum toxin (BT) can be employed as an alternate therapeutic option that uses the chemodenervation approach. The range of dentistry treatment choices is expanding quickly. Applications of non-traditional therapy alternatives, such as the use of BT, are becoming more and more common in this situation. Although BT has been shown to be effective in a number of circumstances, its application in esthetic operations, such as the treatment of facial wrinkles, has gained widespread acceptance. This research is especially interested in applications of BT related to dentistry in the craniofacial region. For many diseases that a dentist would be interested in treating, BT provides a temporary, reversible, and generally safe therapy option. Due to their extensive knowledge of the anatomy of the faciomaxillary region, dental surgeons are a potential pool of operators who, with a small amount of skill enhancement, can use BT in their toolkit. This broadens the scope of minimally invasive alternatives to invasive protocols or refractory conditions. An online search was conducted for the use of BT in dentistry; all studies and articles pertaining to the subject were chosen, and dental-related content was removed and summarized. The fundamentals of BT and some of its applications in dentistry are covered in this article. The comprehensive details of its application in dentistry will be covered in the upcoming sections.

## Introduction and background

The 21st century saw dramatic improvements in the quality of living. The rise of cosmetic solutions in several world regions was among the most significant developments in the medical field. Many individuals have improved as a result of beauty treatments that have an impact on their functional status. Many cosmetological surgeries have also been introduced into the field of dentistry. The use of Botox (Allergan, Inc., Irvine, CA, USA) for beauty procedures is well-reported in the literature and widely covered by the media. Botox, on the other hand, is extracted from several other therapeutic potentials. With its huge trend in dental treatments and advancements in pharmaceutics, there is a comprehension of how Botox can be used to diagnose a range of dentofacial conditions. Many people thought Botox was chiefly used for cosmetic treatments such as facial wrinkles and fine lines; however, botulinum, from which Botox is abstracted, has an ancient legacy of medical applications. Botox is now used more frequently in dental care because of its potential health benefits in the therapies of many oral diseases. The Washington Dental Quality Assurance Commission (DQAC) issued an explanatory comment on July 26, 2013, affirming overall dentists' potential in using Botox and its products for the treatment of structural and cosmetic prerequisites and their direct esthetic effects, provided the dentist has exact, credible training and expertise. Correspondingly, the Michigan Board of Dentistry and the New Jersey State Board of Dentistry have both approved the use of Botox and dermal fillers by dentists [1].

## Review

History

Botulism was first described by Kerner as a life-threatening disease. *Bacillus subtilis*, a gram-positive, rod-shaped, spore-forming anaerobe bacterium, and *Clostridium butyricum* and *Clostridium baratii*, which are commonly found on crops and also in soil, water, and animal fecal matter, produce botulinum toxin (BT) [2]. Botox is one of the deadliest toxic elements used in biological weapons. BT, on the other hand, has two sides. BT was the first to be endorsed for medicinal use. It continued to expand the spectrum of its therapeutic application from Scott's first therapeutic use of BT for strabismus to the present day. Botox is divided into seven types, A through G. Widely viable variants, on the other hand, are filtered exotoxins, with only BT types A (BTA) and B (BTB) being advertised under various brand names [3]. Each Botox vial contains (I) 100 units of *Clostridium botulinum* type A neurotoxin complex; (II) it is a neurotoxin complex; and (III) 0.5 milligrams of human albumin [4].

Mechanism of action

**Figure 1:**
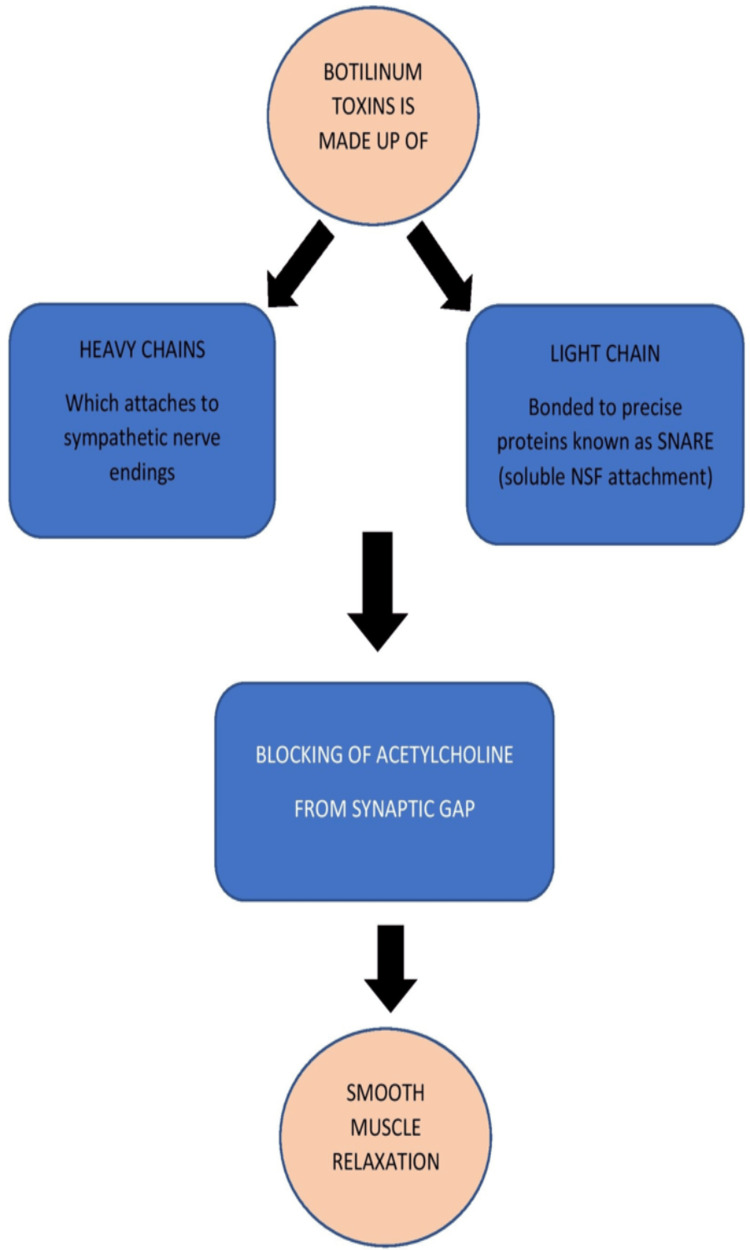
Mechanism of action of Botox The image has been created by the author

BT is responsible for the temporary, concentration-dependent decrease in muscle activity. Additionally, it is genotoxic and relaxes smooth muscle by preventing the release of acetylcholine (ACh) from pain receptors, which causes temporary chemical demyelination of skeletal muscle. The obstruction only lasts for a brief time since new peripheral nerve terminals that are growing will eventually restore the nerve and muscle signal [5]. The use of BT as a treatment is, therefore, more palliative than curative. Additionally, the toxin has been observed to prevent ACh from being released by parasympathetic nerve terminals [6]. BT's mode of action is simple. A heavy and light chain makes up the nerve poison. Chemical reactions happen when substances are introduced into striated muscles. The light chain of the botulinum complex binds to specific proteins called SNARE (soluble NSF attachment protein receptor), blocking ACh adhesion and discharge into the synaptic gap [7]. The heavy chain of the complex attaches to serotonergic sympathetic nerve terminals of the muscles. This prevents glandular pheromones from working properly and temporarily paralyzes muscles. Spasticity, when injected into a muscle, can develop in two to five days and continue for up to three months (Figure 1) [8].

Uses

The term "temporomandibular disorder-synovial joint disorders" (TMD), which Bell developed, covers a wide variety of masticatory system dysfunctions in addition to temporomandibular joint (TMJ) disorders, which are not widely recognized and are usually misunderstood with illnesses associated with chronic pain [9]. The terms TMJ malfunctioning syndrome, operational TMJ disturbances, myofascial pain dysfunction syndrome, and temporomandibular pain dysfunctional syndrome have all been used to describe this set of phenomena [10]. TMD symptoms include facial discomfort, joint crackling, headaches, earaches, neck pain, and/or limited jaw movement. The main etiologic factors of TMD include oromandibular dystonia, external stresses, and cognitive habits. Muscle stiffness and myogenic components linked to bruxism make up the majority of TMD cases [11]. Treatment options for TMD brought on by strong biting forces include surgery, dental restorations, intraoral devices, and occlusion changes. The majority of individuals who undergo these treatments find them to be intrusive, expensive, and irreversible. They might not be able to withstand the parafunctional forces acting continually if we use the most modern approaches for esthetic, conservative repair [12]. Therefore, since Botox produces muscle relaxation in this circumstance, treating such people with it is a very good option. If a muscle relaxant is administered to the masticatory muscles, this gritting reflex can be reduced or even eliminated [13]. Because chewing and swallowing only employ a small portion of the force that is available, a slight reduction in muscle activity can sometimes diminish teeth grinding, but usually not enough to change chewing and deglutition [14]. For individuals who haven't responded to typical therapeutic alternatives, BT injections can provide a therapy that is far less invasive while still delivering the necessary outcomes [15]. With BTX injections, soft tissue activation is suppressed, which lessens muscle tonicity and alleviates symptoms in TMD patients. This inflammatory joint ailment frequently manifests as discomfort and dysfunction, and in more severe cases, it can lead to joint spasticity and muscular atrophy [16]. Chronic arthritic pain is made worse by neuropeptide release in the peripheral area. By modifying the function of nociceptors and alleviating symptoms and the neurogenic inflammatory response, BTX type B prevents the production of neuropeptides [17].

Tooth grinding

The term "bruxism," which refers to the grinding or gnashing of the teeth, is derived from the Greek word "brychein." Tooth wear, periodontal tissue injury, and muscle damage are the causes of the disorder. In numerous studies, it has been discovered that the spasm is triggered when the muscle fibers are not provided with any relief. BTA has been shown to be useful in cases of bruxism. Injections of BTA (with a dosage ranging from 25 to 100 units on each side) into the masseter muscles on both sides have been proven to significantly diminish the severity of symptoms for 6 to 78 weeks (mean 17 to 19 weeks) [17]. For other healthy patients, injections with a concentration of 100 units seem to be dependable [18]. BTs are just as successful at treating bruxism as an oral splint. The use of BTA to treat nighttime teeth grinding is similarly encouraging, with results lasting at least a month after administration [19].

Dental implant and surgery

Lack of osseointegration, which can be brought on by strong masticatory forces in people with aberrant masticatory habits, is the main reason for implant placement failure [20]. Oromaxillofacial fractures necessitate several locations that need to be fixed in order to manage the pressures of the mastication muscles, which decreases the formation of the callus. Prophylactically inject 100 units of BTX type A into both sides of the masseter muscle 12 to 18 hours before surgery to reduce these forces. These injections can reduce the tension in hyperactive muscles in the periodontal apparatus created during periodontal operations [21].

Gummy smile

The "sticky grin," or display of excessive gingival tissue in the upper jaw after smiling, is a problem for maintaining hygiene and carrying out the restorative operation, with no easy solution [22]. It is frequently possible to deduce excessive gum openness from excessive lip muscle tightness. Although the writing has taken into account a few meticulous tactics for the modification of hyperfunctional lip lift muscles, particularly of the top lip, the LeFort-I maxillary bone recontouring with impaction for skeletal vertical maxillary excess, and removal of gingiva for delayed unattached dental emission with an extravagant showcase of the gingiva are largely the most well-acknowledged cautious corrections now used [23,24].

Cancer therapy

Adjuvant BTX type A treatment can improve the tumor response to chemotherapy or radiotherapy by reopening the vascular bed [25]. The toxin is applied locally, improving tumor oxygenation and perfusion, while also regulating vascular vasoreactivity. The best outcomes are observed when the BTX shot is given three days before the initiation of chemotherapy [26,27].

Salivary gland disorders

BTX has been used to treat a number of conditions involving the salivary glands, including sialorrhea, sialocele, and Frey's syndrome. Sialorrhea, or excessive salivation and drooling, is a common condition brought on by unsteady face and mouth muscles [28]. There are many different types of treatment choices, ranging from a preventative medical strategy to a more invasive surgical operation. Investigations have been done into how BTA affects the salivary glands [29]. Drooling can be addressed by administering BTA. The parotid gland receives injections with Botox [30]. BTX increases dopamine levels in the brain, acts as a bronchodilator, and protects sympathetic neurotransmission by activating smooth-muscle adrenoceptors in arteries and veins surrounded by tumors. A dosage of 30 to 70 units reduces saliva flow after four weeks, but the effects wear off after about three months, requiring further injections. It has also been demonstrated that BTA injections have a positive effect on gustatory sweating (Frey's syndrome). Recurring treatment enhances the benefits of the initial treatment [31]. As a surgical complication following parotidectomy, saliva aggregates in the sialoceles, causing tissue response surrounding the saliva. When used in this scenario, BTX type A prevents the secretomotor parasympathetic autonomic nerve from releasing ACh. In the parotid region, subcutaneous doses of 50-70 units are given [32]. Frey's syndrome, commonly referred to as "Gustatory sweating," involves flushing and perspiration of the facial skin during salivary stimulation and mastication. The illness is more likely to develop in patients who have undergone parotid surgery [33]. Since its initial publication in 1995, targeted BTX injections into the area of gustatory sweating have been a well-known therapy option for Frey's syndrome. Regular treatment has been shown to reduce the size of the affected area, and many people can go six months without exhibiting any symptoms [34,35].

Disorders of facial nerves

**Figure 2:**
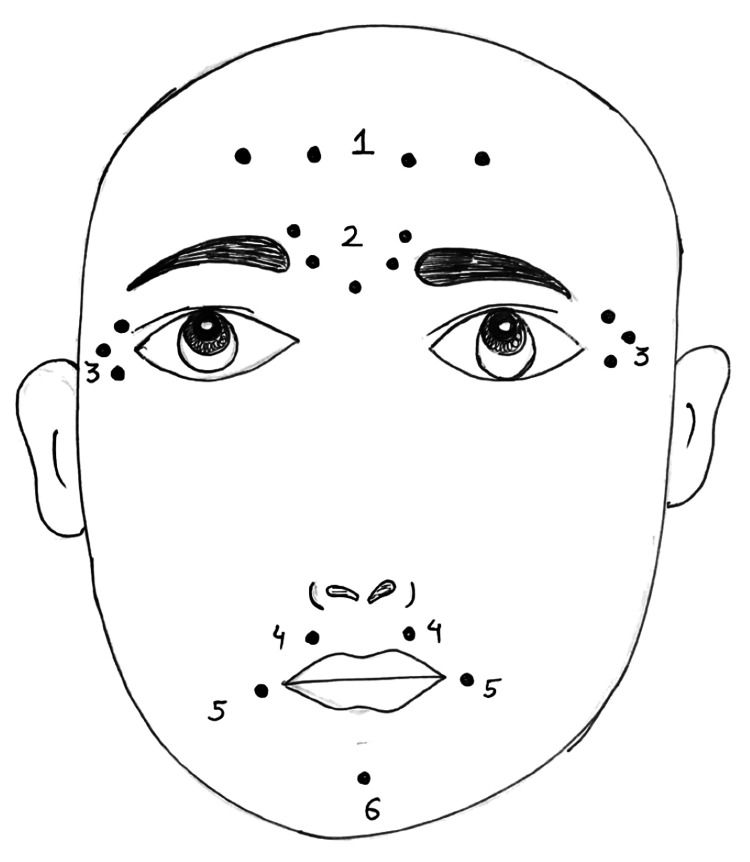
Botulinum toxin type A injection sites on the face 1 - Forehead lines: Frontalis muscle; 2 - Glabellar/frown lines: Corrugator supercilii and procerus muscles; 3 - Crow's feet (lateral orbital lines): Orbicularis oculi; 4 - Perioral lines (smoker's lines), gummy smile: Orbicularis oris muscle; 5 - Marionette lines: Depressor anguli oris; 6 - Mentalis dysfunction: Mentalis muscle The image has been created by the author

By reducing facial synkinesis, BTA injection therapy enhanced facial symmetry and demonstrated symmetrical expression during voluntary movements [36]. Crocodile tears, a sign of facial nerve palsy, are caused by a dysfunctional interaction between the secretomotor fibers of the lacrimal gland and the salivary gland [37]. The injection of BT into the lacrimal gland has been effective in treating this condition. In particular, loss of cervical muscular tone and prolonged facial discomfort brought on by excessive masticatory activity can be effectively and safely treated with BT for maxillofacial pain [38]. The various injection sites of BTA injection are shown in Figure 2. BTA has been shown to be effective in treating trigeminal neuralgia with little adverse effects. BT is fast replacing other invasive procedures as the preferred microsurgical technique for treating trigeminal neuralgia. When injected into precranial muscles, Botox (25-75 units) relaxes overactive muscles by blocking the nerve signals that initiate contractions. According to Elcio, Botox injections can significantly lessen the excruciating pain caused by irritation of the fifth cranial nerve of the face and head [39]. To treat persistent and idiopathic neuralgia, BTX type A suppresses the ACh and other neurotransmitters. BTX type A functions as an analgesic, reducing central and peripheral sensitization by inhibiting neuropeptide discharge from neuropathic neurons. It also lowers noradrenaline and adenosine triphosphate (ATP) discharge from sympathetic nerve terminals. The dosage for treating trigeminal neuralgia is 20-50 units of BTX administered intravenously or into the masseter muscle, according to Zúñiga et al. [40,41]. Recently, resistant cases of postherpetic neuralgia have been treated with BTX Type A. When administered intradermally at 15 units, BTX inhibits the release of substance P, calcitonin gene-related polypeptide (CGRP), and formalin-induced glutamate and has both indirect and direct effects on the sensory and central nervous systems. The discomfort lessens to a reasonable level after the first week of treatment [42].

Trismus

Restricted mouth opening, or trismus, is brought on by dysfunction of the trigeminal nerve. According to this scenario, BTX type A acts at the synaptic terminal of the lower motor neuron that is cholinergic, inducing flaccid paralysis as a result of presynaptic inhibition of neuroexocytosis at the terminal. Trismus is treated with a 25 unit BTX dose administered intramuscularly in each masseter muscle, and a 10 unit dose administered intramuscularly in the temporalis muscle [21].

In wound healing

Hemostasis, inflammation, tissue growth, and remodeling are just a few of the processes that go into the healing of traumatic, surgical, or other wounds (such as fissures and ulcers), each of which, if disrupted, can lead to a chronic illness. During the healing process, there is a contraction of the surrounding muscles, an increase in metabolism, and inflammation [43]. Recently, BTX type A has been used in experimental wound healing due to its capacity to reduce static strain over and around healing tissues. Better esthetics may arise from this chemo-immobilization's capacity to speed up healing and lessen scarring [44]. Treatment for cleft lip and palate is frequently associated with delayed midface development and distorted facial growth. These side effects have been attributed to excessive soft tissue lifting and a tight cheiloplasty, which imposed stress on the healing incision [45]. It has been demonstrated that intraoperative injections of BTX type A into the medial and lateral regions of the orbicularis oris muscle reduce muscular activity, which lowers tension and promotes better healing at the surgical site. Although further research is required to build a standardized approach, this results in the emergence of new trends that utilize BTX in the healing of wounds [46].

Oral vaccine carrier - a while ago, BTX type A expression product was produced using molecular biological techniques. The expression product loses its neurotoxic effects but retains the ability to pass through the gut, enter the bloodstream, and trigger an immune reaction [47]. The toxin passes through a portion of the digestive system after ingestion, moves from the gut lumen to the bloodstream, and in the peripheral nervous system, binds to cholinergic nerve terminals. It is then endocytosed and functions as a metalloendoprotease to cleave important polypeptides for exocytosis. According to Simpson et al., the most important method for entering polarized gut cells is the toxin's specific binding to receptors on that side of the mucosa. The bound toxin is actively transported across cells and delivered intact and unchanged on the serosal side of the monolayer [46,48].

Adverse effects

Possible adverse effects include flu-like symptoms, drooping eyelids or eyebrows, foggy vision, photophobia, indigestion, nausea, sweating, fever, chills, allergic reactions such as rash, itching, dyspnea, tightness of the chest, edema of the face, redness, tingling, bruising, swelling, or soreness at or near the injection site, loss of muscle tone at or near the site, bleeding, restlessness, faintness, lethargy, ringing in the ears, and excessive sweating elsewhere besides the underarms. The two most common medication-related side effects connected with BT dentofacial injections appear to be changes in salivary consistency and dysphagia, as well as trouble in speech and facial muscles [49]. These concerns include injection site-specific and dose-dependent difficulties (lateral pterygoid injections and palatal and tongue muscle injections are more prevalent, for example). When BT effects are detected anywhere other than where it was applied locally, this is known as the "Spread of toxic effect." The orofacial and head-neck region is where this toxic effect can spread the fastest [50,51]. For use during pregnancy, BT is classified in category B, meaning it can be used if the potential benefits outweigh the risks to the developing fetus. Similarly, it is not recommended for use in breastfeeding mothers. The use of BT in children under the age of 18 should be limited, following FDA recommendations for its usage. Botox has no known lethal dose in humans, although injecting around 30 vials, estimated to cost around 3000 U.S. dollars, for dental applications totaling about 80-100 units, has the potential to be fatal [52,53].

Contraindication 

In pregnant women and nursing mothers (not yet fully proven) [54], the use of BTX may impact nerve growth in children (not yet proven). Cases of neuromuscular problems such as Parkinson's disease and myasthenia gravis, which affect hemostasis, are common. In addition, it can pose risks in individuals with certain heart and circulatory problems, pre-existing infection at the injection site, eczema, psoriasis, and other skin illnesses, as well as in those taking aminoglycoside antibiotics, quinine, chloroquine, calcium channel blockers, and aspirin. Emotional disturbances are also noted, especially in individuals over the age of 65 years [1,54].

## Conclusions

The field of dentistry has expanded as a result of BT's metamorphosis from a deadly poison to an extraordinary therapeutic agent. In situations where the patient is unresponsive to, or in conjunction with, less intrusive therapy methods, BT has unquestionably been demonstrated to offer substantial utility in the care of the patient. It offers a minimally invasive method for treating and controlling a limited number of appropriate cases, with the fewest possible problems. However, the dentist in practice must make sure that the procedure falls within his or her area of expertise and that he or she is qualified to handle any possible side effects, in addition to administering it.
